# Accuracy of Allplex SARS-CoV-2 assay amplification curve analysis for the detection of SARS-CoV-2 variant Alpha

**DOI:** 10.2217/fmb-2021-0288

**Published:** 2022-07-26

**Authors:** Tjaša Ž Čretnik, Matjaž Retelj, Sandra Janežič, Aleksander Mahnič, Tine Tesovnik, Robert Šket, Barbara J Bizjan, Samo Jeverica, Mateja Ravnik, Mitja Rak, Mateja Borinc, Maja Rupnik, Tadej Battelino, Marko Pokorn, Jernej Kovač

**Affiliations:** ^1^National Laboratory of Health, Environment & Food, Maribor, Slovenia; ^2^Faculty of Medicine, University of Maribor, Maribor, Slovenia; ^3^Department of Paediatric Endocrinology, Diabetes & Metabolism, University Children’s Hospital, University Medical Centre Ljubljana, Ljubljana, Slovenia; ^4^Faculty of Medicine, University of Ljubljana, Ljubljana, Slovenia

**Keywords:** B.1.1.7, SARS-CoV-2, Seegene SARS-CoV-2 assay, Slovenia, variant of concern

## Abstract

**Aim:** To evaluate the accuracy of two PCR-based techniques for detecting SARS-CoV-2 variant Alpha (B.1.1.7). **Materials & methods:** A multicenter prospective cohort with 1137 positive specimens from Slovenia was studied. A mutation-based assay (rTEST-COVID-19 qPCR B.1.1.7 assay) and amplification curve pattern analysis of the Allplex SARS-CoV-2 assay were compared with whole-genome sequencing. **Results:** SARS-CoV-2 variant Alpha was detected in 155 samples (13.6%). Sensitivity and specificity were 98.1 and 98.0%, respectively, for the rTEST-COVID-19 qPCR B.1.1.7 assay and 97.4 and 97.5%, respectively, for amplification curve pattern analysis. **Conclusion:** The good analytical performance of both methods was confirmed for the preliminary identification of SARS-CoV-2 variant Alpha. This cost-effective principle for screening SARS-CoV-2 populations is also applicable to other emerging variants and may help to conserve some whole-genome sequencing resources.

Since the beginning of the COVID-19 pandemic, more than 1500 variants of the SARS-CoV-2 virus have been characterized. The accumulation of single or multiple mutations by the variant can affect the phenotypic presentation of the variant and alter its transmissibility, infectivity, virulence or antigenicity [1,2]. The latter is of particular importance, because it can directly influence the efficacy of current vaccines and subsequently the success of vaccination programs [3–5]. It has been estimated that a 20% more transmissible variant will replace the current strain mix in approximately 180 days, whereas a 50% more transmissible variant can do so in as little as 74 days. Early detection and genetic characterization of new variants is therefore essential to slow their spread [6,7].

SARS-CoV-2 variant Alpha (B.1.1.7) spread rapidly in the UK in autumn 2020 and quickly became the most prevalent variant in other parts of the world, with a global prevalence of up to 46% [7–9]. Currently, it is still the most common variant in North America and Europe [9] (last accessed 1 August 2021). Variant Alpha is characterized by the presence of 23 mutations across the virus genome, 14 of which encode alterations in the spike protein gene, most notably the substitution in the receptor-binding domain N501Y and two deletions in the N-terminal domain (NTD) ΔH69-V70 and ΔY144 [10,11].

The ideal method for genomic surveillance of SARS-CoV-2 would need to detect all critical mutations and confirm the variant, preferably simultaneously with baseline diagnostics. It would also need to be technically and financially accessible to a sufficient number of laboratories. Genomic changes in a variant virus can alter the detection kinetics of a specific target on RT-PCR (reverse transcription PCR) assays, which also alters the pattern of the amplification curve, which is an assay-specific characteristic [12]. These changes can be used as proxy indicators of the presence of the variant concurrent with virus detection, or they can be used retrospectively to analyze the spread of the variant independent of whole-genome sequencing (WGS) [7,13,14].

In Slovenia, systematic non targeted and targeted genomic surveillance of SARS-CoV-2 was introduced in January 2021 by combining WGS of 10% of all PCR-positive samples and screening of another 10% of PCR-positive samples by specific real-time RT-PCR to detect variant Alpha, respectively. With the increasing number of PCR-positive samples during the third wave of the epidemic and the increasing prevalence of variant Alpha, it became necessary to modify the surveillance protocol. The primary aim of the study was to verify whether the specific real-time RT-PCR used in the targeted approach can be used as a confirmatory test for variant Alpha, as claimed by the manufacturer. At the same time, we evaluated the accuracy of amplification curve pattern analysis of the Allplex SARS-CoV-2 assay as a complementary tool for rapid presumptive identification of variant Alpha in the four laboratories using this assay as the primary diagnostic method for SARS-CoV-2 detection.

## Materials & methods

### Study design

A total of 1139 routine samples positive in the Allplex SARS-CoV-2 assay (Seegene, Seoul, Korea) collected from 1 January to 31 March 2021, were included in the genomic surveillance protocol and analyzed in parallel using the rTEST COVID-19 qPCR B.1.1.7 assay (MultiplexDX, Bratislava, Slovakia) and WGS. For WGS extracted viral RNA from the positive samples was stored at -80 °C until transport and processed in batches once a week by the core laboratory. The study was approved by the institutional review board. The principles outlined in the Declaration of Helsinki were followed for the study and no additional samples were collected for the purpose of the study.

### RT-PCR testing

The Allplex SARS-CoV-2 assay (Seegene, Seoul, Korea) was used as a routine diagnostic RT-qPCR in four clinical microbiology laboratories of the National Laboratory for Health, Environment and Food (NLZOH). In this assay, four sets of primers are used to detect the presence of the E, N, S and RdRP genes of SARS-CoV-2, with the latter two detected on the same fluorescent channel. The results are automatically interpreted by the supplied software Seegene Viewer (Seegene, Seoul, Korea), while the amplification curves can be additionally analyzed visually simultaneously or retrospectively. The assay was performed according to the manufacturer's instructions.

The rTEST COVID-19 qPCR kit was used to detect the B.1.1.7 variant. The assay aims to detects two of the NTD deletions, ΔH69–V70 and ΔY144, characteristic of the B.1.1.7 variant. When these deletions are not present, amplification of the specific B.1.1.7 target versus the consensus target of the rTEST COVID-19 qPCR assay is delayed by at least five cycles, as a recommended cut-off by the manufacturer.

### Amplification curve pattern analysis

**Figure 1.**
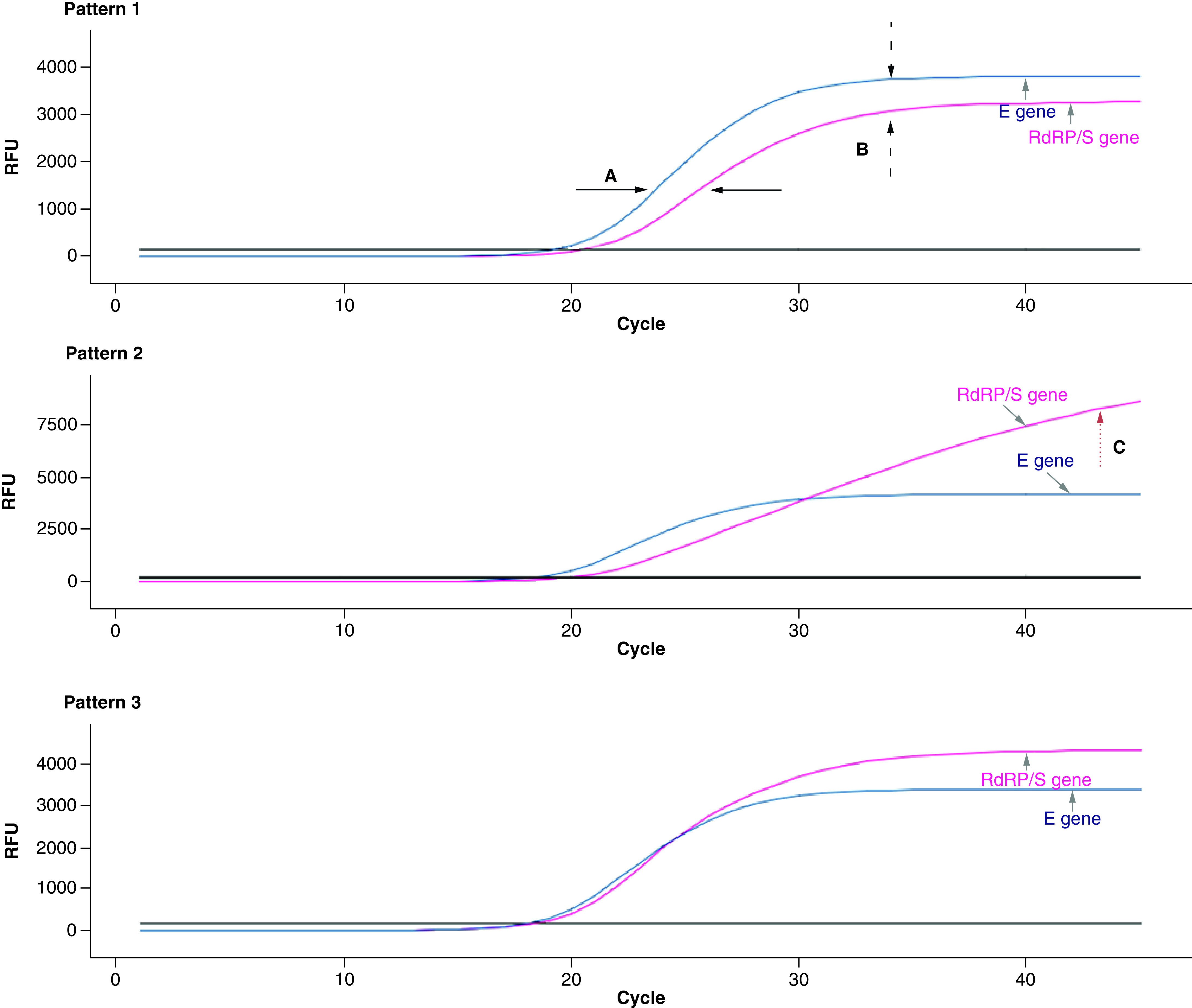
Allplex SARS-CoV-2 assay amplification curve patterns used for preliminary discrimination of Alpha variant. E and S/RdRP gene amplification curves were analyzed. Amplification curve pattern characteristics. Pattern 1: delay between the E and S/RdRP (=RdRP/S) gene curve at the inflection point **(A)** and lower end-point fluorescence in the S/RdRP gene curve compared with E gene curve. **(B)** Pattern 2: sigmoidal E gene and linear S/RdRP gene curve amplification and higher end-point fluorescence in the S/RdRP gene curve. **(C)** Pattern 3: any other curve presentation, the figure shows typical non variant Alpha amplification curves.

The shapes and relative positions of the amplification curves of the E and S/RdRP genes for each sample were subsequently checked in the software Seegene Viewer. The curves were visually analyzed by four different operators, one per laboratory, and classified into three different patterns according to the criteria shown in Figure 1. Briefly, pattern 1 consisted of the delay between the E and S/RdRP gene curve at the inflection point and lower end-point fluorescence in the S/RdRP gene curve compared with E gene curve, pattern 2 consisted of the sigmoidal E gene and linear S/RdRP gene curve amplification and higher end-point fluorescence in the S/RdRP gene curve and pattern 3 consisted of any other curve presentation. Pattern 1 and pattern 2 were considered predictive of variant Alpha. The results of the WGS were blinded to the operators and were revealed after the analysis.

### Whole-genome sequencing

Samples were obtained as RNA extracts and stored at -80 °C until further processing. Libraries were prepared with amplification enrichment using the ARTIC v3 kit according to protocol V.4 with recommended reagents and consumables. Finally, samples were sequenced on either the Illumina MiSeq or NovaSeq platform depending on availability. Consensus genome sequences were obtained by: mapping fastq reads to the reference genome (NC_045512) using BWA aligner (v 0.7.17); 2) implementing the ivar (v 1.3)/samtools (mpileup, v 0.1.19) workflow to filter the quality of aligned reads, remove primer sites and generate the consensus sequence. PANGO lines were assigned using the command-line tool PLEARN (v1.2.13), which allows a maximum of 10% Ns.

### Statistical analyses

To evaluate the diagnostic performance of both the rTEST COVID-19 qPCR B.1.1.7 assay and the Allplex SARS-CoV-2 amplification curve pattern analysis in detecting variant Alpha, the sensitivity, specificity, positive predictive value (PPV) and negative predictive value for each method were calculated using WGS as a reference. A receiver operating characteristic curve was generated to verify the cut-off value set by the manufacturer of the rTEST qPCR COVID-19 kit. Analysis in GraphPad Prism (GraphPad Software, CA, USA) resulted in a table of possible cut-off values and their respective sensitivities and specificities with 95% CIs compared with WGS. The cut-off value with the highest Youden’s index (sensitivity + specificity - 1) was considered optimal. A p-value of 0.05 was considered statistically significant. Predictive values were determined using GraphPad QuickCalcs (GraphPad Software, CA, USA). Agreement of results between amplification curve pattern analysis and WGS was assessed using Cohen’s kappa statistics with the following interpretive guidelines: no agreement (0.00), low agreement (0.00–0.20), fair agreement (0.21–0.40), moderate agreement (0.41–0.60), substantial agreement (0.61–0.80) and near perfect agreement (0.81–1.00). The analysis was performed using GraphPad QuickCalcs.

## Results

A total of 17 variants of SARS-CoV-2 were characterized among 1139 RT-PCR-positive samples (Table 1). Variant B.1.258.17 was detected in 896 samples (78.8%) and variant B.1.1.7 in 155 samples (13.6%). Of the 155 confirmed variant Alpha positive samples by WGS, 151 were positive by Allplex SARS-CoV-2 amplification curve pattern analysis (97.4%) and 152 were also positive by the rTEST COVID-19 qPCR B.1.1.7 kit (98.1%). We detected 959 (98.0%) true negative results using the amplification curve pattern analysis and the rTEST qPCR COVID-19 B.1.1.7 kit yielded 964 (97.5%) true negative results.

**Table 1.** The number and proportion of SARS-CoV-2 variants among sequenced samples in Slovenia, January–March 2021 (n = 1139).

Variant	n	%
B.1.258.17	896	78.7
B.1.1.7 (variant Alpha)	155	13.6
B.1.258	20	1.8
B.1.160	17	1.5
B.1.1.70	11	1.0
B.1.149	9	0.7
B.1.177	8	0.7
B.1	6	0.5
B.1.221	5	0.4
B.1.2	3	0.3
B.1.36	2	0.2
B.1.525	2	0.2
B.1.159	1	0.1
B.1.1.58	1	0.1
B.1.139	1	0.1
B.1.1.282	1	0.1
A	1	0.1
Total	1139	100

The receiver operating characteristic curve to determine the optimal cut-off value of the rTEST qPCR assay is shown in the Supplementary Material. This value represents the maximum number cycles for which the amplification of the B.1.1.7 target on the S gene is delayed compared with the consensus target in a variant Alpha sample. The area under the curve was 0.9833 (95% CI: 0.9692–0.9975; p < 0.0001). The cut-off of 5.1 cycles was selected and the evaluation of the performance of rTEST qPCR assay was based on this cut-off value.

The sensitivity, specificity, PPV and negative predictive value using WGS as reference are shown in Table 2. Predictive values were calculated based on the prevalence of 13.6% relevant throughout the observation period and the prevalence of 49.5% calculated during the peak prevalence of variant Alpha in March 2021.

**Table 2.** Diagnostic performance of Allplex SARS-CoV-2 amplification curve pattern analysis compared with rTEST COVID-19 qPCR B.1.1.7 kit for the detection of SARS-CoV-2 variant Alpha.

Test characteristic	Allplex SARS-CoV-2 assay (amplification curve pattern analysis) % (95% CI)	rTEST qPCR assay % (95% CI)	Combination of both assays % (95% CI)
Sensitivity	97.4 (93.5–99.3)	98.1 (94.5–99.5)	95.5 (90.9–98.2)
Specificity	97.5 (96.3–98.4)	97.9 (96.8–98.6)	99.2 (98.4–99.7)
Positive predictive value (13.6% prevalence)	85.8 (80.4–89.9)	88.4 (83.1–92.2)	94.8 (90.2–97.3)
Negative predictive value (13.6% prevalence)	99.6 (98.9–99.8)	99.7 (99.1–99.9)	99.3 (98.6–99.7)
Positive predictive value (49.5% prevalence)	97.4 (96.2–98.2)	97.9 (96.8–98.7)	99.1 (98.3–99.6)
Negative predictive value (49.5% prevalence)	97.4 (96.4–98.3)	98.1 (94.4–99.4)	95.7 (91.6–97.9)

The agreement of the results between the amplification curve pattern analysis and the WGS was satisfactory when the results of all participating NLZOH laboratories were considered (Table 3).

**Table 3.** Agreement between the Allplex SARS-CoV-2 amplification curve pattern analysis results and whole-genome sequencing results.

NLZOH^†^ location	WGS results	Allplex SARS-CoV-2 amplification curve analysis results		Kappa	(95% CI)
		Pattern 1 and 2	Pattern 3		
A	B.1.1.7	80	1	0.96	(0.93–1.00)
	Non-B.1.1.7	4	280		
B	B.1.1.7	25	3	0.73	(0.61–0.86)
	Non-B.1.1.7	13	283		
C	B.1.1.7	27	0	1.00	(1.00–1.00)
	Non-B.1.1.7	0	196		
D	B.1.1.7	19	0	0.81	(0.68–0.94)
	Non-B.1.1.7	8	200		
Total	B.1.1.7	151	4	0.90	(0.86–0.93)
	Non-B.1.1.7	25	959		

† National Laboratory for Health, Environment and Food, Slovenia.

WGS: Whole-genome sequencing.

## Discussion

For rapid determination of the specific SARS-CoV-2 variant, several screening protocols can be used based on the detection of nonspecific changes in diagnostic PCR target signals or on the detection of specific mutations associated with a particular viral variant. Failed positive spike gene signals have been evaluated and used in several countries facing rapid spread of variant Alpha (B.1.1.7) [12,13,15,16]. However, gene amplification failure may have other causes, and this approach is less specific than PCR assays designed to detect a specific mutation or set of mutations. Here, we show good analytical performance of both approaches for variant Alpha with way over 97% sensitivity and specificity.

Although the two most common lineages present in our sample B.1.258.17 and B.1.1.7 share one of the two NTD deletions that the rTEST q PCR COVID-19 B.1.1.7 kit is designed to detect, namely ΔH69-V70, we have shown that the assay is still able to distinguish between these variants and detect B.1.1.7 with 98% specificity at the selected cut-off value. To our knowledge, no other independent evaluation of this assay has been published to date.

As additional variants of concern and variants currently under investigation have been introduced in Slovenia in the last 6 months, screening for only one of these variants was no longer a sufficient approach. The observation that the curve of the S/RdRp genes deviated from the expected shape of the curve, leading to possible prescreening of variant Alpha, was reported in the technical report of the manufacturer of the Allplex SARS-CoV-2 assay in February 2021 and later published by Italian authors [13]. In only four samples in this study, the presence of variant Alpha was confirmed by WGS. The authors therefore concluded that visual analysis of amplification curve profiles could be used mainly as a tool for retrospective studies.

In addition to the previously described delay in S/RdRP amplification, we found linearization of the S/RdRP gene amplification curve to be a separate and equally important change. Although, the interpretation criteria were descriptively defined, the combination of both patterns proved to be 97% sensitive and specific compared with WGS. Different analysts independently obtained satisfactory agreement of results with WGS in four different laboratories.

Finally, one of the advantages of systematic genomic monitoring performed using WGS is also the ability to compare other laboratory methods and procedures to the gold standard. High-quality evaluation of the analytical performance of diagnostic tests and procedures allows laboratories to adjust their surveillance and diagnostic protocols and respond in a timely manner to new epidemiological circumstances.

There are several limitations in our study. First, we were unable to determine the underlying molecular mechanism for the change in amplification curves; it is most likely due to a lower efficiency of the PCR reaction. Second, the specificity of such a nonspecific screening necessarily depends on the biological background of variants in a given geographic area, and cannot necessarily be extrapolated to other geographic locations. Third, following the rapid evolution of SARS-CoV-2 variants, the clinical utility of our results may be limited to areas where variant Alpha is still present, and to laboratories using a particular diagnostic assay that was also used in our situation. Fourth, there were some differences between sites in kappa. This can be explained by geographic variations of the virus, possible coinfection with different variants or by the subjective nature of the pattern analysis itself. Nevertheless, we report here the largest evaluation of its kind with more than 1000 SARS-CoV-2 genomes completely sequenced.

## Conclusion

We describe here a good analytical performance of the two methods for the preliminary identification of SARS-CoV-2 variant Alpha. They can both be used as screening tests and even as confirmatory tests in areas with high prevalence of this lineage. Considering the still high prevalence of this variant in several regions and an increase in the prevalence of other variants of concern, similar approach can be used to monitor the prevalence of known SARS-CoV-2 variants in a cost-effective manner and save some WGS resources.

## Future perspective

Genomic surveillance of the SARS-CoV-2 pandemic is important for planning, implementation and analysis of containment measures. Cheaper and faster approaches are needed for surveillance, especially in resource-limited settings. Simultaneous detection of the virus and typical variants is a promising and feasible approach, considering that the major circulating variants in a geographical area are known.

**Summary points**

- The accuracy of two PCR-based techniques for the detection of variant Alpha (B.1.1.7) in a large multicenter prospective cohort in Slovenia from January to March 2021 with 1137 positive samples were evaluated.
- A specific mutation-based assay (rTEST COVID-19 qPCR B.1.1.7 assay) and nonspecific curve pattern analysis of the Allplex SARS-CoV-2 assay were compared with whole-genome sequencing.
- Variant Alpha was detected in 155 samples (13.6%). Sensitivity and specificity were high for both assays, 98.1 and 98.0% for the specific assay and 97.4 and 97.5% for the nonspecific analysis, respectively.
- The positive predictive value of both tests was 97.4 and 97.7%, respectively, with a prevalence of 49.5%.
- A good analytical performance of both methods was confirmed for the preliminary identification of SARS-CoV-2 variant Alpha and both can be used as screening and even as confirmatory tests in areas with high prevalence of this lineage.
- This principle of cost-effective way to screen SARS-CoV-2 populations is also applicable to other emerging variants and may help to conserve some whole-genome sequencing resources.
