# Supplementary material for: Accuracy of Allplex SARS-CoV-2 assay amplification curve analysis for the detection of SARS-CoV-2 variant Alpha
Source: Future Microbiol. 2022 Jul 26:10.2217/fmb-2021-0288. doi: 10.2217/fmb-2021-0288 (PMC9332907; doi:10.2217/fmb-2021-0288)
Supplement: Supplementary file 1 [file Supplementary_material.docx]

# Supplementary material


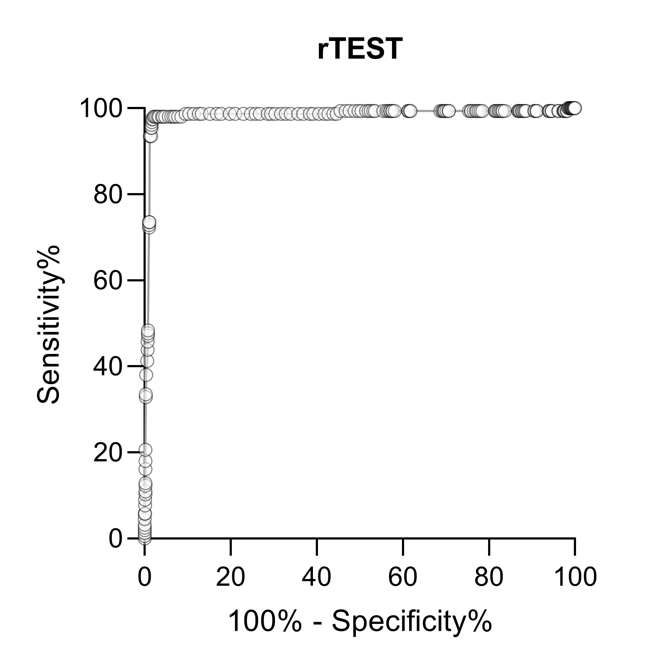


**Supplementary Fig. 1:** The receiver operating characteristic (ROC) curve discriminating differences in Cq values determined in the rTEST COVID-19 qPCR B.1.1.7 kit of B.1.1.7 lineage and non-B.1.1.7 lineage samples


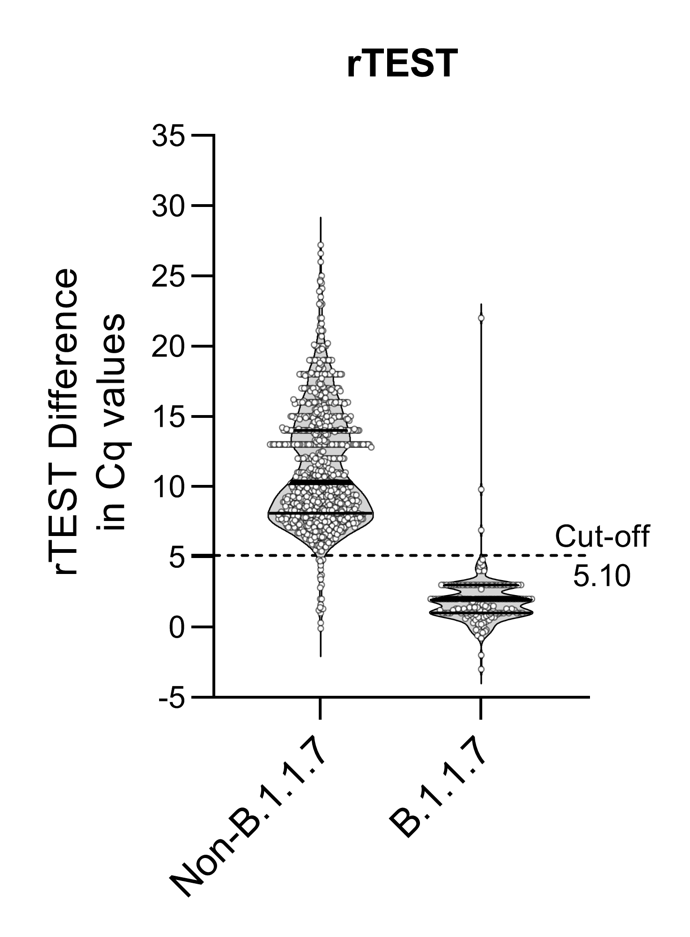


**Supplementary Fig 2:** Violin plots with median (solid thick lines), quartiles (thin lines) and individual Cq differences (open circles) determined in the rTEST qPCR assay for B.1.1.7 lineage and non- B.1.1.7 lineage samples

**Supplementary table 1:** The performance of different cut-offs from the receiver operating characteristic (ROC) analysis of the rTEST COVID-19 qPCR B.1.1.7 kit

| Cut-off | Sensitivity | | Specificity | | Youden index |
| --- | --- | --- | --- | --- | --- |
|  | % | (95% CI) | % | (95% CI) |  |
| < 4.65 | 97.4 | (93.6-99.0) | 98.3 | (97.3-98.9) | 194.7 |
| < 4.75 | 97.4 | (93.6-99.0) | 98.1 | (97.0-98.8) | 194.5 |
| < 5.10 | 98.1 | (94.5-99.5) | 97.9 | (96.8-98.6) | 194.9 |
| < 5.45 | 98.1 | (94.5-99.5) | 97.7 | (96.5-98.4) | 194.7 |
| < 5.60 | 98.1 | (94.5-99.5) | 97.6 | (96.4-98.4) | 194.6 |
